# Liposomal Delivery of a Biotechnological *Lavandula angustifolia* Miller Extract Rich in Rosmarinic Acid for Topical Herpes Simplex Therapy

**DOI:** 10.3390/antiox14070811

**Published:** 2025-06-30

**Authors:** Federica Fulgheri, Fabrizio Angius, Matteo Perra, Ilenia Delogu, Silvia Puxeddu, Milen I. Georgiev, Renáta Novotná, Jana Franková, Misia Lobina, Aldo Manzin, Maria Manconi, Maria Letizia Manca

**Affiliations:** 1 Department of Life and Environmental Sciences, University of Cagliari, 09042 Monserrato, Italy; federica.fulgheri92@unica.it (F.F.); misia.lobina@unica.it (M.L.); manconi@unica.it (M.M.); mlmanca@unica.it (M.L.M.); 2 Department of Biomedical Sciences, Section of Microbiology and Virology, University of Cagliari, 09042 Cagliari, Italy; fangius@unica.it (F.A.); deloguilenia@gmail.com (I.D.); silvia.px@live.com (S.P.); aldomanzin@unica.it (A.M.); 3 Laboratory of Metabolomics, Department of Biotechnology, Institute of Microbiology, Bulgarian Academy of Sciences, 4002 Plovdiv, Bulgaria; milengeorgiev@gbg.bg; 4 Department of Plant Cell Biotechnology, Center of Plant Systems Biology and Biotechnology, 4000 Plovdiv, Bulgaria; 5 Department of Medical Chemistry and Biochemistry, Faculty of Medicine and Dentistry, Palacký University Olomouc, 775 15 Olomouc, Czech Republic; renata.novotna@upol.cz (R.N.); frankova0@seznam.cz (J.F.)

**Keywords:** cutaneous delivery, drug delivery system, antioxidant, antiviral activity, nanocarriers, Normal Human Dermal Fibroblasts, Vero cells

## Abstract

Herpes simplex virus type 1 (HSV-1) is a widespread pathogen responsible for recurrent infections, primarily affecting the skin and mucous membranes. With the aim of targeting both the viral infection and the associated inflammatory response, biotechnologically produced *Lavandula angustifolia* Miller (*L. angustifolia*) extract, rich in rosmarinic acid, was incorporated into liposomal formulations intended for topical application. Lavender is known for its strong anti-inflammatory, antioxidant, wound-healing, and antiviral properties. However, its low stability under certain conditions limits its therapeutic potential. Four different formulations were developed: conventional liposomes, glycerosomes, hyalurosomes, and glycerohyalurosomes. The vesicles were characterized for size, stability, and entrapment efficiency. Glycerosomes were the smallest (~58 nm), while the other formulations ranged around 77 nm, all maintaining a highly negative surface charge, ensuring stability and reduced aggregation. Glycerol-containing formulations demonstrated superior stability over 12 months, while liposomes and hyalurosomes increased their size after only two months. Entrapment efficiency reached up to 100% for most vesicles, except for glycerohyalurosomes (~54%). In vitro studies on Normal Human Dermal Fibroblasts (NHDFs) demonstrated that all formulations were biocompatible and enhanced cell viability under oxidative stress. Glycerosomes, hyalurosomes, and glycerohyalurosomes exhibited significant anti-inflammatory activity by reducing MMP-1 and IL-6 levels in LPS-stimulated fibroblasts. Furthermore, these preliminary results highlighted promising antiviral activity against HSV-1 of the obtained formulations, particularly when applied during or post-infection. Overall, these phospholipid vesicles offer a dual therapeutic approach, combining antioxidant, anti-inflammatory, and antiviral effects, positioning them as promising candidates for the treatment of HSV-induced skin lesions and related inflammatory conditions.

## 1. Introduction

The Herpes simplex virus (HSV), an enveloped double-stranded DNA virus and part of the Herpesviridae family, has afflicted humanity for thousands of years, leaving an indelible mark on human health, infecting its hosts with long-lasting life effects [1]. The two types, HSV-1 and HSV-2, are mainly responsible for human infections, each of them with distinct epidemiological and clinical characteristics [2]. The prevalence of HSV infections is alarming, with around 67% of the population under the age of 50 (3.7 billion) infected by HSV-1, which is mostly associated with oral lesions [3]. On the other hand, it is estimated that almost 520 million people aged 15–49 (around 13%) are infected by HSV-2, the main cause of genital herpes, contributing to a substantial burden of sexually transmitted infections [2,4]. Following primary infection at the entry site, the viral lytic replication results in a cytopathic effect that leads the host cell to death, jeopardizing the tissue integrity. The main clinical manifestation of HSV infections is the formation of painful blisters and ulcers, especially in mucous membranes [5]. The virus can spread through direct contact with wounds or infected body fluids [6]. These infections are not limited to a specific anatomical site, because both viruses can infect several tissues and organs, leading to oral, ocular, neurological, and systemic diseases [7].

Additionally, HSV remaining latent within the host cells causes persistent infections for the entire life of the host, and it can evade the immune system [8]. Stress, illness, UV light exposure, and other factors may cause the viral reactivation from latency, often resulting in recurrent episodes of disease [9]. HSV has also been associated with a range of complications, like neonatal herpes, encephalitis, and increased susceptibility to other sexually transmitted infections [8,10,11].

Despite being among the most prevalent viral infections worldwide, HSV infections still represent a significant challenge to medical science and public health. To date, there is no approved cure for HSV infection, and the available treatments are limited to the symptoms [12]. Approved antiviral medications (e.g., acyclovir, ganciclovir) primarily reduce viral replication and symptom duration but do not eliminate the virus or prevent latency reactivation [12]. Moreover, drug resistance and systemic toxicity further limit their long-term efficacy [13,14]. Consequently, there is a pressing need for novel topical agents that can address both viral activity and tissue damage.

In recent years, the research has provided unprecedented insights into the biology of HSV, sparking renewed efforts to uncover novel strategies for prevention and treatment [15].

Thanks to their low side effects and consolidated uses over the years in traditional medicine, natural products and extracts have garnered increasing attention for their therapeutic applications [16]. *Lavandula angustifolia* (*L. angustifolia*), commonly known as lavender, has been extensively studied due to its rich phytochemical profile and associated biological activities. Lavender is rich in terpenes, flavonoids, and phenolic acids [17]. Several studies have demonstrated the strong antioxidant activity of lavender extracts or essential oils, largely attributed to high concentrations of phenolic compounds, such as rosmarinic acid, isoferulic acid, luteolin, and caffeic acid derivatives [17,18,19]. Moreover, growing evidence suggests that lavender extracts also exhibit antiviral activity, particularly against enveloped viruses like HSV-1 [20,21,22]. Among these bioactive compounds, rosmarinic acid stands out due to its well-documented antioxidant and anti-inflammatory effects [23,24].

**Figure 1:**
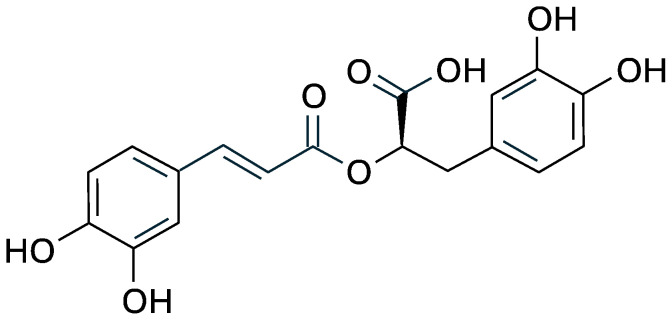
Structural formula of rosmarinic acid.

Chemically, rosmarinic acid is an ester of caffeic acid and 3,4-dihydroxyphenyllactic acid, and its chemical structure includes two catechol groups (Figure 1) that confer high antioxidant capacity by donating hydrogen atoms to free radicals and chelating metal ions involved in oxidative reactions [25,26].

Because of these properties, rosmarinic acid has been widely studied for its therapeutic potential against several diseases, such as psoriasis, arthritis, atherosclerosis, and herpes infections [27,28,29].

Recent advancements in biotechnology have enabled the production of high yield extracts from *L. angustifolia* cell suspensions, offering an alternative to the traditional extraction methods [30]. These innovative approaches allow for the selective enrichment of specific bioactive molecules, particularly rosmarinic acid. For instance, Koycheva et al. reported a rosmarinic content of approximately 100.16 mg/g in an extract obtained from cell cultures, as confirmed by HPLC and NMR analyses [29]. This is notably higher than the concentrations typically found in flower extracts of *L. angustifolia*, where rosmarinic acid concentrations are reported in the range of 829.68–1229.33 µg/g. This highlights the potential of biotechnological approaches to obtain enriched extracts with significantly higher concentrations of target phenolic compounds. Taken together, these bioactivities, along with the extract tolerance profile, make *L. angustifolia* a promising candidate for topical delivery in the treatment of viral and inflammatory skin conditions.

However, despite its significant therapeutic potential, *L. angustifolia* extracts, rich in rosmarinic acid and many other phenolic compounds, face challenges commonly associated with natural products, such as high chemical instability, poor permeability, and, consequently, low bioavailability, which limit its efficacy in both cosmetic and pharmaceutical applications [31,32,33,34].

Over the years, several methods based on nanotechnology have been used to overcome these limitations. Among them, liposomes and liposome-like vesicles have demonstrated the ability to protect sensitive compounds from degradation, enhance their solubility, and facilitate targeted delivery to specific tissues [35,36,37]. Notably, liposome-like vesicles modified with glycerol or sodium hyaluronate (called glycerosomes and hyalurosomes, respectively) are able to enhance skin penetration and dermal delivery of various bioactive compounds [38,39,40,41].

Considering these previous findings, with the aim of targeting both the viral infection and the associated inflammatory response, a biotechnologically produced *Lavandula angustifolia* Miller extract, rich in rosmarinic acid, was incorporated into four types of lipid-based vesicles. Conventional liposomes were used as a control, while glycerosomes, hyalurosomes, and glycerohyalurosomes were designed to evaluate the effects of glycerol and/or sodium hyaluronate on vesicle properties and efficacy.

The obtained vesicles were characterized in terms of size, surface charge, entrapment efficiency, and stability over time. Their biocompatibility and ability to protect cells from oxidative damage and inflammatory responses were assessed using Normal Human Dermal Fibroblasts (NHDFs). Finally, the antiviral and virucidal effects of *L. angustifolia* extract alone or loaded in the aforementioned vesicles were evaluated against the cytopathic effect related to the HSV-1 infection.

## 2. Materials and Methods

### 2.1. Materials

The Lipoid S 75 (S75) was purchased from Lipoid AG (Cologne, Germany) with the support of its Italian agent AVG srl (Milan, Italy). Methanol, ethanol, and all other products with analytical grade were purchased from Sigma-Aldrich (Milan, Italy). Medium, fetal bovine serum, penicillin, streptomycin, and the other reagents and plastics, unless otherwise specified, used for in vitro cell culture studies were supplied by Thermo Fisher Scientific Inc. (Waltham, MA, USA). Water was purified through a Milli-Q system from Millipore (conductivity: 18.2 MΩ cm at 25 ± 1 °C; Milford, MA, USA).

### 2.2. Preparation and Chemical Characterization of Lavandula angustifolia Miller Extract

*Lavandula angustifolia* Miller extract was biotechnologically produced as previously reported [30]. Briefly, *L. angustifolia* cell suspension was incubated at 26 ± 1 °C, using Linsmayer and Skoog (LS) as the growth medium, supplemented with sucrose (30 g/L) and 2,4-dichlorophenoxyacetic acid (0.2 mg/L), in the dark and under constant agitation (100 rpm). The biomass was then collected, dried, and treated with a mixture of water and ethanol (50:50 *v*/*v*) in an ultrasonic bath for 20 min at room temperature (25 ± 1 °C) to ensure complete extraction of the targeted compounds. The resulting extractive solution was filtered, concentrated under vacuum at 40 ± 1 °C, lyophilized, and stored at −20 ± 1 °C prior to use. The dry extract powder was analyzed by means of high-performance liquid chromatography (HPLC) and nuclear magnetic resonance (NMR) imaging as previously described [29].

### 2.3. Antioxidant Activity of Lavandula angustifolia Miller Extract

The antioxidant activity of the *L. angustifolia* extract was measured as a function of its ability to scavenge l-2,2-diphenyl-1-picrylhydrazyl (DPPH), a stable free radical characterized by an intense purple-red color, which turns yellow when scavenged by an antioxidant molecule. The methanolic solution of the extract (10 µL) was diluted (1:100) with a methanolic solution of DPPH (40 µg/mL), the diluted samples were kept at room temperature and in the dark for 30 min, then the absorbance of the solution was measured at 517 nm, using a microplate reader (Synergy 4, BioTek Instruments, AHSI S.p.A, Bernareggio, Italy). The antioxidant power of the samples was calculated according to the formula below:

$$\mathrm{Antioxidant Activity}\%=[({\mathrm{ABS}}_{\mathrm{DPPH}}-{\mathrm{ABS}}_{\mathrm{sample}})/{\mathrm{ABS}}_{\mathrm{DPPH}}]\times 100$$

### 2.4. Preparation of Vesicles

The composition of the vesicular formulations was selected based on previous studies reporting their efficiency in delivering phytochemicals and ensuring nanosystem stability [42,43]. Four different formulations were prepared (Table 1), each of them containing 20 mg/mL of *L. angustifolia* extract and 120 mg/mL of S75, differing in the dispersing medium used. Liposomes were obtained using water, glycerosomes using a mixture of glycerol and water (50:50 *v*/*v*), hyalurosomes using a sodium hyaluronate solution at 0.2% *w*/*v*, and glycerohyalurosomes using a mixture of glycerol and sodium hyaluronate solution at 0.4% *w*/*v* (50:50 *v*/*v*). The obtained dispersions were immediately sonicated (13 µm, 20 cycles, 5 s on and 2 s off) using a Soniprep 150 ultrasonic disintegrator (MSE Crowley, London, UK) to obtain small and homogeneous vesicles. Each formulation was prepared in triplicate and independently repeated in at least three separate batches.

### 2.5. Characterization of Vesicles

Freshly prepared samples were diluted 1:100 in water and analyzed using a Zetasizer Ultra (Malvern Instrument, Worcestershire, UK) at 25 ± 1 °C to determine the mean diameter and polydispersity index (PI) through the dynamic light-scattering method. Zeta potential was measured using the M3-PALS (Mixed Mode Measurement-Phase Analysis Light Scattering) technique, which measures the electrophoretic mobility of particles.

Unentrapped phytochemicals were removed by means of dialysis. Briefly, 1 mL of each sample was placed into a Spectra/Por^®^ dialysis tube (12–14 kDa MW cut-off, 3 nm pore size; Spectrum Laboratories Inc., Breda, The Netherlands) and purified for 2 h at room temperature under constant stirring in 2 L of water, which was refreshed after 1 h. The entrapment efficiency (EE%), i.e., the percentage of bioactives that are successfully entrapped into vesicles, was calculated as the percentage of the concentration of phytochemicals after dialysis versus the concentration before dialysis. The concentration of phytochemicals was measured as a function of the antioxidant activity of the dispersions with the DPPH assay, as described in Section 2.2.

Samples were stored at 4 ± 1 °C for 12 months. To evaluate their stability over time, each month mean diameter, polydispersity index, and zeta potential were measured.

### 2.6. Cell Viability and Protection Against Oxidative Stress

Normal Human Dermal Fibroblasts (NHDFs) were isolated from skin fragments. The skin specimens were obtained from healthy tissue taken from plastic surgery patients from Faculty Hospital Olomouc after informed consent. The study was performed according to the Code of Ethics of the World Medical Association (ref. number 41/09). These cells were chosen to respect the future potential of selected substances for wound healing. NHDFs were cultured in Dulbecco’s Modified Eagle’s Medium (DMEM) supplemented with 10% (*v*/*v*) fetal bovine serum and antibiotics (penicillin (100 mg/mL) and streptomycin (100 U/mL)), at 37 °C with 100% humidity and 5% CO_2_. The medium was changed every 72 h. The NHDFs between 2–3 passages were used for the experiments [44].

NHDFs were seeded in 96 well plates at the final density of 1 × 10^4^ cells/mL. The following day, the tested samples (*L. angustifolia* extract dispersion, liposomes, glycerosomes, hyalurosomes, and glycerohyalurosomes), in a concentration range from 0.04 µg/mL to 40 µg/mL in serum-free DMEM, were applied to the cells for 24 h. After incubation period, cell viability was assessed using the MTT assay, which measured the mitochondrial reduction of MTT (3-(4,5-Dimethylthiazol-2-yl)-2,5-diphenyltetrazolium bromide) to formazan at 540 nm.

The in vitro protective effect of the formulations against cell damages caused by oxidative stress was evaluated as well. The NHDFs were seeded into 96-well plates at a density of 1 × 10^4^ cells/mL. After 24 h of incubation, the cells were stressed with hydrogen peroxide (250 µM) and simultaneously treated for 1 h with sub-toxic concentration (40 µg/mL) of *L. angustifolia* extract, in dispersion or loaded into vesicles, diluted in serum-free DMEM medium. Untreated NHDFs were used as control (100% viability). NHDFs untreated and stressed with hydrogen peroxide (250 µM; for 1 h) were used as the positive control. After incubation, the NHDFs were washed with phosphate buffered saline (PBS, pH 7.4), and their viability was determined by the MTT assay. The results are expressed as the percentage of control (100% viability). NHDFs were carefully washed with PBS after both the treatment phase and the incubation with MTT to ensure the complete removal of residual formulation components.

### 2.7. Scratch Assay Infected by Lipopolysaccharide

For scratch assay, the NHDFs were seeded in 6-well plates at a cell density of 0.5 × 10^5^ cells/mL and cultivated until reaching confluence. When the NHDFs reached the confluence, the lipopolysaccharide (LPS, *Pseudomonas aeruginosa*, final concentration 10 µg/mL) was added to half of the plates for 6 h to stimulate the pathological inflammatory response [45]. Next, all plates with NHDFs (included plates with LPS) were scratched by using a 5 mL pipette to produce a wounded area. The culture medium was removed and tested samples (*L. angustifolia* extract water solution or formulations) were applied (in a concentration range from 4 µg/mL to 40 µg/mL) in serum-free DMEM for 24 h. At the end of the incubation period, the cell culture medium was collected and stored at −80 ± 1 °C. The following three controls were used: (1) NHDFs untreated and not scratched as negative control (C); (2) NHDFs untreated and scratched as positive control (CS); (3) NHDFs untreated and scratched that were stressed with LPS (CL). Levels of interleukin-6 (IL-6), matrix metalloproteinase-1 (MMP-1) and matrix metalloproteinase-2 (MMP-2) in the cell supernatant after the treatment were examined using ELISA Human IL-6 Kit (900-K16), Human Total MMP-1 DuoSet ELISA (DY901B-05) and Human MMP-2 Duo-Set ELISA (DY902) (R&D Systems, Minneapolis, MI, USA) according to the manufacturer’s protocol.

### 2.8. Antiviral Activity Tests

**Figure 2:**
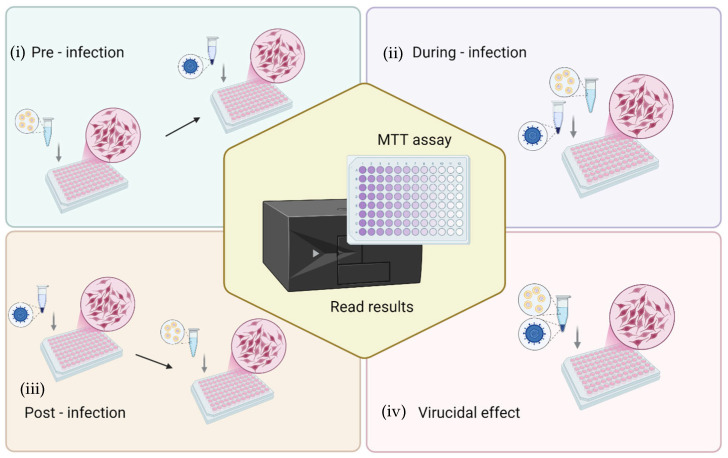
Schematic representation of the experimental setups for evaluating the protective effect of *L. angustifolia* formulations against the cytopathic effect of HSV-1 in Vero cells. Four experimental conditions were tested: (i) pre-infection (cells treated with formulations prior to viral exposure), (ii) during-infection (formulations and virus added simultaneously), (iii) post-infection (cells treated with formulations after viral infection), and (iv) virucidal effect (virus pre-incubated with formulations before cell infection). After treatment and infection, cell viability was determined by the MTT assay to quantify protection against virus-induced cytopathic effects.

The antiviral activity of formulations was evaluated by the cytopathic effect assay, which is based on infecting cell cultures and measuring the ability to protect cells from virus-induced cytopathic effects by cell viability. In brief, African green monkey kidney (Vero) cells (ATCC collection, Manassas, VA, USA) were grown in DMEM (Corning Inc., Corning, NY, USA) supplemented with 10% fetal bovine serum (FBS), 1% penicillin/streptomycin and incubated at 37 °C with 100% humidity and 5% CO_2_. Cells were seeded in 96-well plates at a density of 3.0 × 10^4^ cells/well and, after 24 h of incubation, were processed for antiviral activity testing against HSV-1 strain KOS (ATCC VR-1493). Four different challenging experiments were performed to investigate the step of the viral cycle in which the formulations exerted action, as follows: (i) pre-infection: the cell monolayer was first treated with formulations for 3 h, then washed and viral suspension added for the viral absorption time; (ii) during-infection: formulations and virus at multiplicity of infection (MOI) of 0.01, were added simultaneously to the cell monolayer; (iii) post-infection: the cell monolayer was first infected with the virus for 1 h and then the cells were washed and treated with formulations; (iv) finally, to verify the virucidal effect, the viral suspension was incubated with the formulations for 1 h at room temperature; thereafter, this suspension was added to the cells for 1 h. The experimental setups used to assess the protective effect of formulations against HSV-induced cytopathic effects are shown in Figure 2. In each experiment, after the viral adsorption, the cells were washed with PBS, and fresh culture medium was added. After 72 h incubation, the cells were washed with PBS, and 450 μM MTT was added to each well. After 2–3 h, cells were washed with PBS and the formazan crystals were dissolved in DMSO, and the optical density was measured at 570 nm with a microplate reader (Tecan Infinite 200, Männedorf, Switzerland) [46,47]. All the experiments were repeated at least three times and in duplicate. The results are presented as the percentage of protection from viral cytotoxic effect as calculated by comparing the viability observed in treated samples vs. the infected control cells and/or mock infected untreated cells. Moreover, to discriminate the formulation concentration-associated cytotoxicity from the infection-related cytopathic effect, the cytotoxicity of the formulations on Vero cells and the percent of cell viability of uninfected treated cells vs. untreated cells were also evaluated. Finally, the half-maximal effective concentration (EC50) of the formulations was inferred by curve-fitting interpolation and compared to the reference compound (acyclovir).

### 2.9. Statistical Analysis

Results are expressed as the means ± standard deviations. Multiple comparisons of means (ANOVA) were used to substantiate statistical differences between groups, while Student’s *t*-test was used to compare two samples. Significance was tested at the 0.05 level of probability (p). Data analysis was carried out with the software GraphPad Prism v10 (GraphPad Software, San Diego, CA, USA) for macOS.

## 3. Results

### 3.1. Extract Characterization

The biotechnological extract was obtained from *L. angustifolia* cell suspension culture capable of producing rosmarinic acid [30]. The high amount of rosmarinic acid ((R)-a-[[3-(3,4-dihydroxyphenyl)-1-oxo-2E-propenyl]oxy]-3,4-dihydroxy-benzenepropanoic acid) in the dry extract powder was previously confirmed by means of HPLC and NMR, with HPLC analysis revealing a content of ~100.16 mg/g extract in the cell suspension [29].

The antioxidant activity of *L. angustifolia* extract at a final concentration of 200 µg/mL, (~20 µg/mL of rosmarinic acid), quantified with the DPPH assay, was ~80%.

### 3.2. Vesicle Preparation and Characterization and Stability

The physico-chemical properties (mean diameter, polydispersity index, and zeta potential) of vesicular systems enriched with glycerol, sodium hyaluronate, or with the combination of both additives, were studied and compared with those of conventional liposomes used as reference (Table 2).

The particle size of liposomes, hyalurosomes, and glycerohyalurosomes was ~77 nm in diameter. The use of glycerol alone as a co-solvent has led to smaller vesicles (~58 nm). The addition of additives has influenced the system in terms of homogeneity, as liposomes had the lowest polydispersity index (0.16), which increased up to 0.27 when both glycerol and sodium hyaluronate were used. Surface charge was highly negative in all vesicles, especially in the case of glycerosomes, hyalurosomes, and glycerohyalurosomes. In all cases, the vesicles were suitable for topical administration.

The entrapment efficiency (EE), calculated as the percentage of antioxidant activity of vesicle dispersions measured before and after removal of unentrapped phytochemicals, is remarkably high for liposomes, glycerosomes, and hyalurosomes, consistently equal to or greater than 94%. In the case of glycerohyalurosomes, the combination of glycerol and sodium hyaluronate ensures the formation of a viscous and structured system capable of retaining lower but substantial quantities of bioactive compounds contained in the extract, such as rosmarinic acid (EE = ~54%).

**Figure 3:**
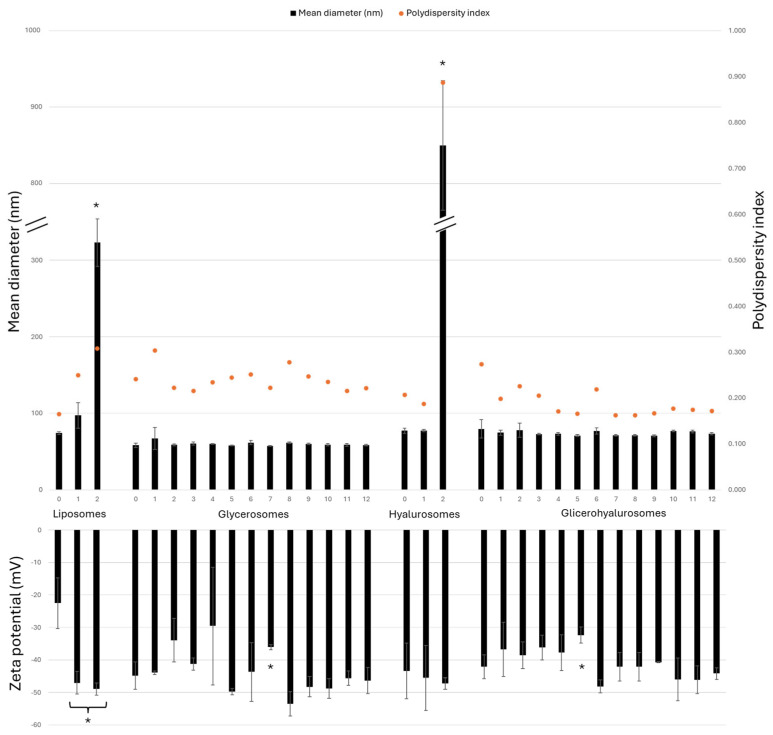
Mean diameter (nm), polydispersity index, and zeta potential (mV) values of *Lavandula angustifolia* Miller extract loaded vesicles stored for 12 months at 4 ± 1 °C. Data are reported as mean values ± standard deviations (error bars) (*n* = 3). * *p* < 0.05 were considered significant compared to corresponding freshly prepared formulations (0 months).

To confirm the stability of the vesicular formulations prepared during the study, they were stored at 4 ± 1 °C for a period of 12 months and evaluated for their mean diameter, polydispersity index, and zeta potential each month (Figure 3). Conventional liposomes, as well as hyalurosomes, experience a significant increase in size after 2 months, as the sizes are 4 times and 11 times larger, respectively, compared to the sizes of the corresponding freshly prepared formulations. As shown in Figure 2, glycerosomes and glycerohyalurosomes displayed remarkable stability, as their sizes did not statistically change throughout the storage period (~60 and ~74 nm, respectively).

On the other hand, the zeta potential remains highly negative for all tested formulations, with no big variations observed during the 12-month storage.

### 3.3. Cell Viability and Protection Against Oxidative Stress

The cytotoxicity of formulations on primary NHDFs was evaluated using the MTT test (Figure 3). The MTT test is a well-established, reliable, and widely accepted method for evaluating cell viability in response to liposomal formulations. Roggia et al. evaluated the possible interference between liposomes and MTT. They found that the spectra for liposomes were similar to the controls, indicating that there is no interference, thus showing reliability of the results obtained through this assay [48].

All the formulations were highly biocompatible, irrespective of the concentration. Cell viability was always higher than 70%, therefore the compounds are not cytotoxic to NHDFs according to ISO 10993-5:2009. Moreover, the treatment with liposomes, glycerosomes, and hyalurosomes at the highest concentration of *L. angustifolia* Miller extract (40 µg/mL) exerted a proliferative effect as the viability of primary NHDFs was ~120%.

**Figure 4:**
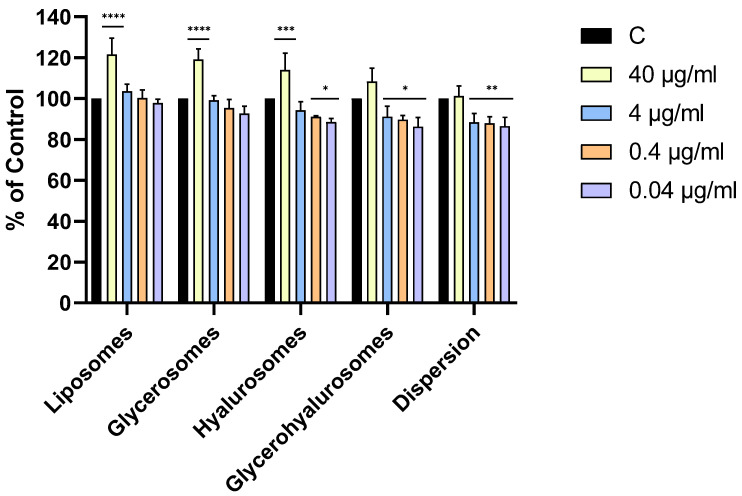
Viability of NHDF treated with *L. angustifolia* extract in dispersion or loaded in liposomes, glycerosomes, hyalurosomes, and glycerohyalurosomes. Untreated NHDFs were used as a control (C). Results are expressed as a percentage of control viability and SD. Number of measurements: *n* = 3. * *p* < 0.05; ** *p* < 0.01; *** *p* < 0.001; **** *p* < 0.0001 were considered significant compared to control (C).

The ability of the formulations to protect NHDF from oxidative stress caused by hydrogen peroxide was evaluated as well (Figure 4). The viability of NHDF not treated, stressed with hydrogen peroxide was ~70%. On the contrary, when the cells were exposed to hydrogen peroxide and treated with formulations loading Lavandula angustifolia Miller extract, the viability was significantly increased, reaching almost 100%. These results confirmed the ability of the obtained vesicles to counteract the formation of peroxide radicals, thus avoiding cell damage and death.

### 3.4. Scratch Assay: Determination of MMP-1, MMP-2 and IL-6

Matrix metalloproteinases (MMPs) and cytokines (ILs) are involved in the regulation of the healing process and play an important role in immune response and tissue repair.

**Figure 5:**
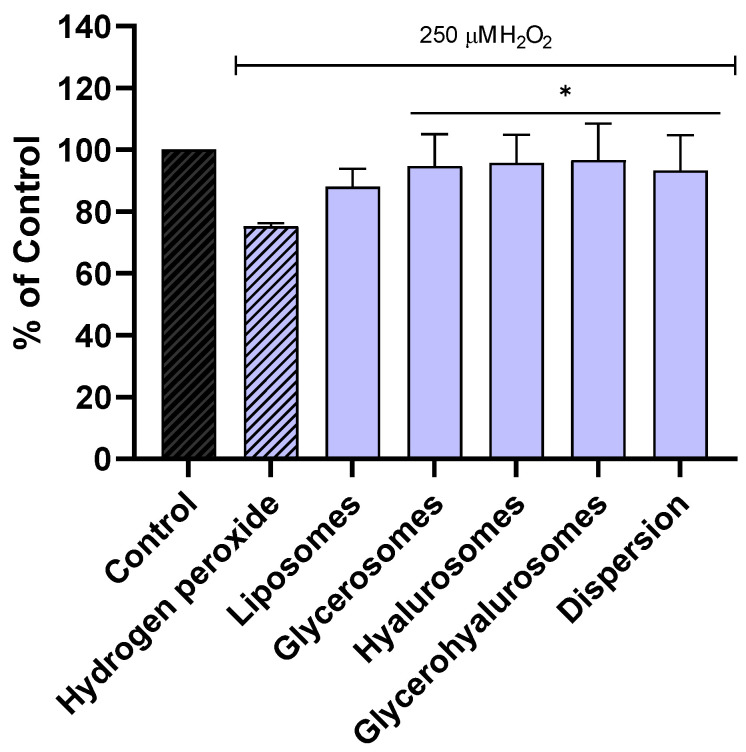
Viability of NHDFs stressed with hydrogen peroxide and treated with *L. angustifolia* extract in dispersion or loaded in liposomes, glycerosomes, hyalurosomes, and glycerohyalurosomes. Untreated NHDFs were used as a control (C). Results are expressed as a percentage of control viability and SD. Number of measurements: *n* = 4. * *p* < 0.05 were considered significant compared to hydrogen peroxide.

ELISA was used to determine levels of the MMP-1, MMP-2, and IL-6 released into the cultivation medium after scratch assay and treatment with extract loaded vesicles. Based on the MTT test results, two concentrations were selected: 4 µg/mL and 40 µg/mL (Figure 5).

**Figure 6:**
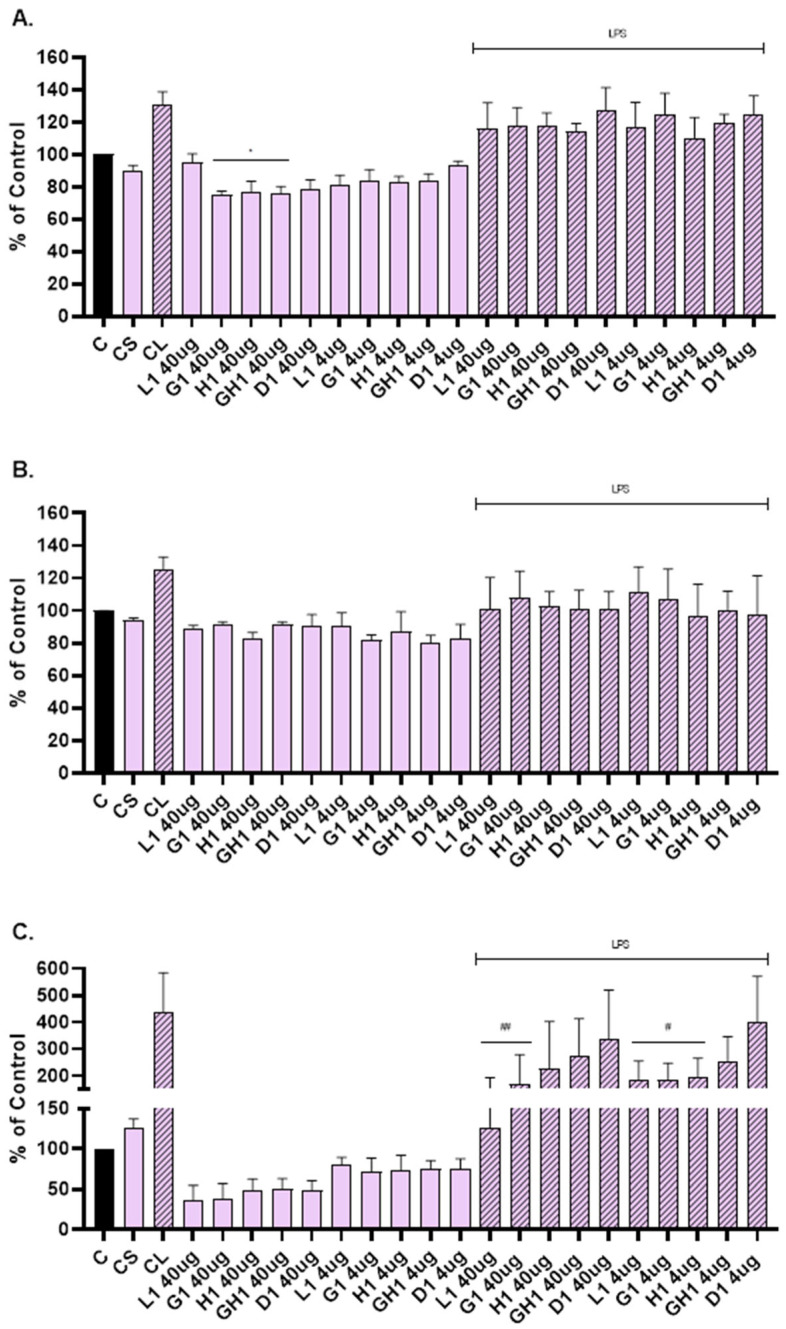
Effect of *L. angustifolia* extract loaded liposomes (L1), glycerosomes (G1), hyalurosomes (H1), and glycerohyalurosomes (GH1) on MMP-1 (**A**), MMP-2 (**B**), and IL-6 (**C**) production by primary scratch NHDF. The control (C) was cells that were not scratched and treated. The other controls were untreated scratched cells (CS) and untreated scratched cells with LPS (CL). Number of measurements: *n* = 3. The symbol * indicates statistical significance with *p* < 0.05 vs. cells that were not scratched and treated (C). The symbol # indicates statistical significance with *p* < 0.05 vs. untreated scratched cells with LPS (CL). The symbol ## indicates statistical significance with *p* < 0.01 vs. untreated scratched cells with LPS (CL).

Vesicles were able to suppress the level of MMP-1 (Figure 6A), MMP-2 (Figure 6B), and IL-6 (Figure 6C) compared to the cells that were not scratched and treated (C). The level of MMP-1 was significantly reduced when NHDFs were treated with glycerosomes, hyalurosomes, and glycerohyalurosomes at a concentration of 40 µg/mL (Figure 6A). Liposomes, glycerosomes, and hyalurosomes also significantly reduced the level of pro-inflammatory IL-6 compared to LPS-stimulated control (CL), (Figure 6C).

### 3.5. Cytotoxicity on Vero Cells

**Figure 7:**
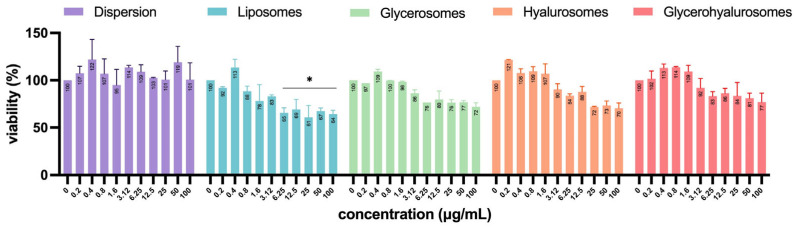
Viability of Vero cells treated with *L. angustifolia* extract in dispersion or loaded in liposomes, glycerosomes, hyalurosomes, and glycerohyalurosomes. The symbol * indicates statistical significance with *p* < 0.05 vs. untreated infected control (0 µg/mL) as calculated by two-way ANOVA and Bonferroni’s post-hoc test.

Vero cells are a well-established model for studying viral infections and are commonly used in antiviral and virucidal assays due to their high susceptibility to various viruses, including HSV-1 [49,50,51]. To exclude any formulation-associated toxic effects that might confound interpretation of infection-related cytopathic outcomes, the cytotoxicity of formulations at different concentrations (0–100 µg/mL) was also evaluated on Vero cells (Figure 7). Almost all formulations did not have significant cytotoxicity, as the percentage of cell viability was not significantly different from untreated control (100%). Despite a progressive decrease in viability with increasing concentration being observed for all the tested formulations, only the treatment with high concentrations of *L. angustifolia* extract loaded liposomes (from 6.25 up to 100 µg/mL) determined a significant cytotoxic effect, as Vero cells’ viability decreased up to ~35%.

### 3.6. Antiviral Activity Against HSV-1

**Figure 8:**
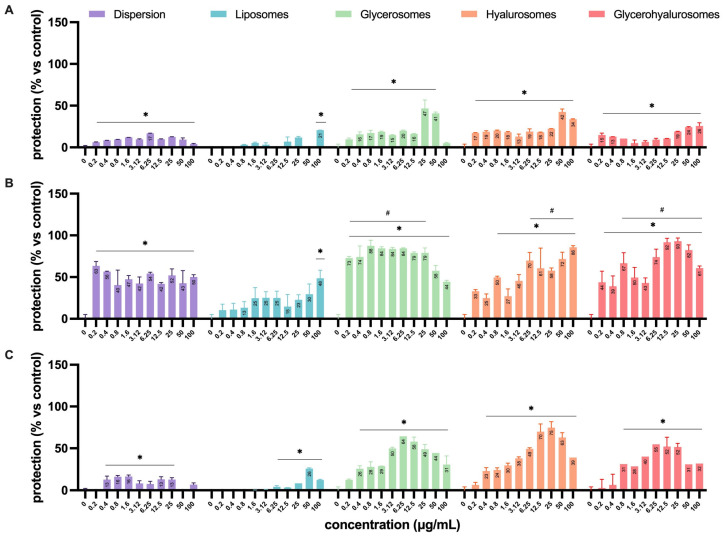
Antiviral activity against HSV-1 of *L. angustifolia* extract in dispersion or loaded into formulations at different concentrations and in the different experimental setups: (**A**) pre-infection, (**B**) during-infection, and (**C**) post-infection formulation treatments. The symbol * indicates statistical significance with *p* < 0.05 vs. untreated and infected control (0 µg/mL). The symbol # indicates statistical non-significance vs. untreated and uninfected control (100%), as calculated by two-way ANOVA and Bonferroni’s post-hoc test.

The antiviral activity was assessed by means of four different experimental configurations (Figure 2) that allowed us to define in which step of the virus life cycle the formulations exert their antiviral effect (Figure 8). The experiments performed by treating Vero cells with the formulations before the infection (Figure 8A) demonstrated a lower capacity to protect the cell monolayer from the cytopathic action of HSV-1 when compared to the simultaneous treatment during the infection (Figure 8B) or the treatment after the infection (Figure 8C). In particular, when cells were treated before the infection, the dispersion had a significant protective effect on cells from a concentration of 0.4 up to 50 µg/mL, with a percentage of protection of ~17%.

When *L. angustifolia* extract was loaded into liposomes, it was able to exert a significant protective effect only at the highest concentration (100 µg/mL), with a percentage of protection that reached ~20%. On the contrary, the treatment with glycerosomes, hyalurosomes, or glycerohyalurosomes determined a protective effect irrespective of the concentration, with a percentage of protection ranging from ~17% to ~50% (Figure 8A).

When Vero cells were treated with formulations simultaneously with the infection (during infection), the *L. angustifolia* extract in dispersion, or loaded into glycerosomes and glycerohyalurosomes, exerted a significant protection against the cytopathic effect induced by HSV-1, irrespective of the concentration, with a percentage of protection close to 100%. Similarly, hyalurosomes were able to protect the Vero cells from HSV-1 at a concentration range of 0.8 up to 100 µg/mL, also reaching a percentage of protection of ~100%. In contrast, extract-loaded liposomes were able to exert a protective effect only at the highest concentration, 100 µg/mL (Figure 8B).

Finally, when cells were treated with the formulations after the infection, the *L. angustifolia* extract in dispersion exerted a significant protective effect at concentrations ranging from 0.4 to 25 µg/mL. The treatment with extract-loaded liposomes determined a significant protection in the range of concentrations from 6.25 to 100 µg/mL, with a percentage of protection of ~30% at 50 µg/mL. On the contrary, the post-infection treatment with *L. angustifolia* extract loaded glycerosomes or hyalurosomes exerted a significant protective effect at all tested concentrations, with the only exception being 0.2 µg/mL. If compared to the two previous formulations, glycerohyalurosomes exerted a slightly lower protective activity, at concentrations ranging from 0.8 to 100 µg/mL. All three formulations reached a percentage of protection of ~80%. For these last three formulations, it is interesting to note how some concentrations, even the lowest for glycerosomes (0.2 µg/mL), were so effective as to reach a protection comparable to 100% (Figure 6).

**Figure 9:**
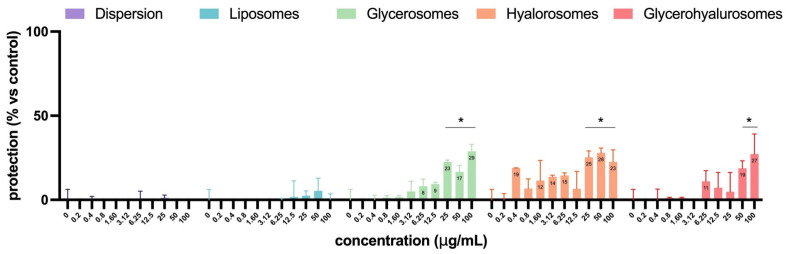
Virucidal effect of *L. angustifolia* extract in dispersion or loaded into formulations at different concentrations. The symbol * indicates statistical significance with *p* < 0.05 vs. untreated and infected control (0 µg/mL), as calculated by two-way ANOVA and Bonferroni’s post-hoc test.

In Figure 9, the virucidal effect of *L. angustifolia* extract in dispersion or loaded into formulations is reported. The extract in dispersion or loaded into liposomes did not have any virucidal activity. Instead, glycerosomes, hyalurosomes, and glycerohyalurosomes were able to exert virucidal activity at higher concentrations, from 25 up to 100 µg/mL for glycerosomes and hyalurosomes and 50 and 100 µg/mL for glycerohyalurosomes (Figure 9). These results suggest that the antiviral activity observed is mainly attributable to substances activated following cellular metabolism rather than to their direct action on HSV-1.

The antiviral efficacy of *L. angustifolia* extract, in dispersion or loaded into formulations, against HSV-1 infection was compared to acyclovir by measuring EC50 values in pre-infection, during infection, and post-infection conditions (Table 3). During infection, both the dispersion and glycerosomes exhibited the highest antiviral activity with EC50 values below 0.20 µg/mL, significantly outperforming acyclovir (9.80 µg/mL). Glycerohyalurosomes and hyalurosomes also demonstrated strong antiviral effects during infection, with EC50 values of 0.59 and 1.79 µg/mL, respectively. Pre-infection treatment showed moderate prophylactic efficacy for glycerosomes (23.82 µg/mL) and hyalurosomes (53.76 µg/mL), whereas acyclovir was ineffective. Post-infection, glycerosomes, hyalurosomes, and glycerohyalurosomes maintained antiviral activity with higher EC50, ranging from 3.53 to 5.76 µg/mL, if compared to acyclovir (1.91 µg/mL). Liposomes were the least effective formulation across all conditions.

## 4. Discussion

In this study, a biotechnologically produced *L. angustifolia* extract, rich in rosmarinic acid, has been loaded into phospholipid vesicles, specifically tailored for topical administration. The extract exhibited ~80% antioxidant activity, measured with DPPH assay at a final concentration of 200 µg/mL, corresponding to approximately 20 µg/mL of rosmarinic acid based on its quantified content (100.16 mg/g).

Similar results were observed by Aldoghachi et al. for pure rosmarinic acid. The authors observed that the antioxidant activity of pure rosmarinic acid, at a concentration of 25 µg/mL, was ~89% [52]. Moreover, the overall antioxidant capacity of the extract (88 µmol TE/g) falls in the lower range compared to values reported in the literature for similar extracts (104.58–206.77 µmol TE/g) [53]. These differences may be attributed to variations in extraction method, plant source, and the relative abundance of other phenolics or synergistic compounds. Despite this, the extract retained a consistent and measurable antioxidant effect, supporting its potential as a reliable component in functional formulations. 

Four different formulations were obtained: conventional liposomes that were used as control; glycerosomes, characterized by a high glycerol content (10 to 50%) and a more flexible phospholipid membrane that improves penetration and transport of bioactives into the deeper layers of the skin [54,55]; hyalurosomes, innovative phospholipid vesicles modified with sodium hyaluronate, which improves skin permeation by promoting the therapeutic activity of the incorporated active ingredients [43,56]; and finally, glycerohyalurosomes, characterized by the combination of both glycerol and sodium hyaluronate, and capable of further enhancing the effect of the active ingredients incorporated at the skin level [57,58].

The first phase of this study was focused on the characterization of the vesicular systems by evaluating the possible influence of composition on the properties of the vesicles and their suitability for topical administration in terms of size, stability over time, and ability to incorporate high amounts of *L. angustifolia* extract.

Glycerosomes were the smallest vesicles (~58 nm) with a homogeneously monodisperse system (0.24), indicating that glycerol ensures good packing of phospholipid molecules, thus increasing curvature, resulting in smaller vesicles. This observation is consistent with previous findings by Manca et al., who reported that glycerosomes loaded with diclofenac had smaller diameters compared to conventional liposomes, with improved deformability and skin penetration properties [54]. Liposomes, hyalurosomes, and glycerohyalurosomes were slightly larger in size (~77 nm), with polydispersity index values consistently below 0.3 (0.16, 0.21, and 0.27, respectively), indicating narrow size distribution and good homogeneity, an essential characteristic for reproducible dermal application. Furthermore, with the exception of liposomes (~−23 mV), all the vesicles were characterized by a strongly negative surface charge of ~−43 mV. A surface charge of ±30 mV is generally associated with stable nanodispersions due to sufficient electrostatic repulsion, thus reducing the phenomena of vesicle aggregation and fusion [59,60]. These parameters confirm the physico-chemical stability of the prepared systems and their suitability for topical delivery.

It appears that glycerol has played a significant role in maintaining the stability of vesicles, as the formulations containing it (glycerosomes and glycerohyalurosomes) have proven to be the most stable over time, with their sizes remaining unchanged over a period of 12 months. This might be due to the ability of glycerol to increase the viscosity of the solution, thus exerting a positive effect on vesicles’ stability over time [54]. In contrast, for liposomes and hyalurosomes, it was only possible to measure their sizes up to 2 months. Similar results were reported by other authors [54,58,61]. For example, Manca et al. observed that glycerosomes were highly stable during the considered storage period, irrespective of the concentration of glycerol (10, 20, and 30%), while conventional liposomes underwent a significant increase of the mean diameter, which almost doubled, from ~81 nm at the beginning to ~180 nm after 1 month of storage [54]. Regarding hyalurosomes, Casula et al. reported the same stability issues, as after three months of storage the authors reported that the main physico-chemical characteristics of the obtained *Zingiber officinalis* extract loaded hyalurosomes become undetectable [61].

Regarding the entrapment efficiency, when both glycerol and sodium hyaluronate were used, the system was able to load lower amount of payload (EE = ~54%) when compared to liposomes, glycerosomes, and glycerohyalurosomes, which were able to load up to 100% of payload.

The in vitro evaluation of toxicity and efficacy represent one of the major factors to be considered when characterizing new molecules or delivery systems [62,63]. Dermal fibroblasts, one of the main cell types of the dermis, have proven to be one of the major players involved in the wound healing process [64]. For this reason, in this work, the biocompatibility of the obtained vesicular systems and their ability to counteract hydrogen peroxide-induced oxidative stress were evaluated in vitro, using Normal Human Dermal Fibroblasts (NHDFs). All formulations of *L. angustifolia* extract in the concentration range of 0.04–40 µg/mL were sub-toxic for primary NHDFs. Moreover, the concentration of 40 µg/mL of extract loaded into liposomes, glycerosomes, and hyalurosomes significantly increased the viability of primary NHDFs, up to ~120% (*p* < 0.05 versus control). Results agree with those previously reported by other authors for the biocompatibility of natural compounds loaded vesicles modified with glycerol and/or hyaluronic acid [57,58]. Furthermore, glycerosomes, hyalurosomes, and glycerohyalurosomes exhibited remarkable protective effects when NHDFs were stressed with hydrogen peroxide. These results demonstrated that the obtained formulations did not alter the ability of rosmarinic acid to inhibit both hydrogen peroxide oxidative damages and hydrogen peroxide-induced inflammatory response in NHDFs [65].

Both lavender extracts, particularly essential oils, and rosmarinic acid have proven to be able to exert strong anti-inflammatory and wound-healing effect both in vitro and in vitro [66,67,68,69,70]. Considering this, the ability of the *L. angustifolia* extract, in dispersion or loaded into the vesicles, to inhibit the production of inflammation mediators was evaluated in vitro on fibroblasts stressed with lipopolysaccharide (LPS), a toxic substance capable of triggering the cascade reactions characteristic of the inflammatory process [71]. Glycerosomes, hyalurosomes, and glycerohyalurosomes at a concentration of 40 µg/mL were the most effective in reducing the MMP-1 level. The level of IL-6 was most reduced by liposomes and glycerosomes (40 µg/mL), which significantly reduced IL-6 levels in LPS-stimulated NHDFs.

All formulations were tested at subtoxic concentrations (0.04–40 µg/mL), within which no pro-inflammatory or pro-oxidant effects were observed. These results are in line with previous findings reporting the anti-inflammatory activity of *L. angustifolia* extracts in different cell models, including macrophages and monocytes [72,73]. However, bell-shaped dose–response relationships have been described for various plant-derived polyphenols, including rosmarinic acid, where low concentrations exert protective effects, while higher doses may lead to paradoxical pro-oxidant or cytotoxic responses [74,75,76]. Moreover, studies have shown that the anti-inflammatory potency of *L. angustifolia* may vary depending on the flowering stage and phytochemical composition [72]. These considerations highlight the importance of dose optimization and extract standardization in the development of safe and effective topical formulations.

The involvement of matrix metalloproteinases (MMPs) in wound healing is well documented, as MMPs play a crucial role in extracellular matrix regulation, both for the inflammatory phase and tissue remodeling [77]. In particular, excessive MMP activity has been associated with delayed wound healing and increased tissue damage in various pathological conditions, including chronic wounds [78].

Lesions caused by HSV infection, similarly to others, are characterized by tissue damage and an intense inflammatory response [79]. Yang et al. demonstrated that following induction of keratitis in Balb/c mice by inoculating the cornea with HSV-1 (KOS strain), MMP-2 and MMP-9 expression significantly increased in the epithelium compared to uninfected controls, with localization in the superficial stromal tissue and inflammatory cells beneath the epithelium [80].

To further explore the potential of our formulations, we assessed their antiviral activity against HSV-1 through different experimental setups, allowing us to determine at which stage of the viral life cycle they exert their effect. Acyclovir, a well-established antiviral therapy, was used as reference to provide a benchmark for evaluating the efficacy of the tested formulations. Acyclovir remains the gold standard for HSV therapy, acting as a potent and selective inhibitor of viral DNA polymerase, and being highly effective in reducing HSV replication and lesion duration [81,82]. However, its use is limited by a lack of anti-inflammatory or wound-healing effects, the emergence of resistant viral strains (especially in immunocompromised patients), and, in systemic applications, potential renal and neurological toxicity [83].

Results revealed that pre-infection treatment of cells with the formulations provided limited protection against the virus-induced cytopathic effect. However, when the formulations were applied simultaneously with infection or post-infection, a significantly stronger protective effect was observed. Glycerosomes and hyalurosomes exhibited the highest antiviral activity, with protection reaching nearly 100% during infection and up to 80% post-infection, even at low concentrations. Interestingly, despite a slightly lower entrapment efficiency, glycerohyalurosomes exhibited high antiviral activity as well. This might be due to a synergistic effect of glycerol and sodium hyaluronate, which contribute to enhanced skin interaction, bioadhesion, and cellular uptake. In fact, glycerol has been shown to increase the deformability and fluidity of vesicle bilayers, facilitating their passage through the stratum corneum and improving dermal delivery of bioactive compounds [54,84]. Moreover, its humectant and anti-irritant properties support skin hydration and barrier function, creating a more favorable microenvironment for antiviral efficacy. Sodium hyaluronate, on the other hand, confers strong bioadhesive properties that prolong vesicle residence time on the skin and enable sustained release. It also interacts with CD44 receptors expressed on keratinocytes and fibroblasts, promoting endocytosis and intracellular delivery of the encapsulated extract [85,86]. In contrast, liposomes and *L. angustifolia* extract dispersion displayed a more limited effect, requiring higher concentrations to achieve moderate levels of protection. Finally, when compared with acyclovir, the obtained formulations exhibited notable antiviral activity against HSV-1, with EC50 values markedly lower than that of acyclovir. In particular, with the only exception of liposomes, they demonstrated superior efficacy during infection, with EC50 values below 1.79 µg/mL, compared to 9.80 µg/mL for acyclovir.

The antiviral activity observed in the obtained vesicular formulations loaded with *L. angustifolia* extract are in agreement with previous research describing the efficacy of lavender-derived compounds against HSV-1. Several studies have demonstrated the antiviral properties of *Lavandula* species, including *L. angustifolia* and *L. austroapennina*, which showed significant inhibition of HSV-1 replication in vitro [20,22]. In particular, lavender stem extract, rich in oleanolic acid, was able to completely inhibit viral replication at low concentrations, especially when administered prior to viral adsorption [22]. Additionally, an essential oil, rich in linalyl acetate and linalool, obtained from *L. angustifolia* exhibited promising antiviral effects against H5N1 influenza virus replication [87]. Considering both the results obtained in this work and previous findings, the antiviral effect is likely the result of multiple constituents acting through different pathways. Rosmarinic acid, reported as a major component, may play a key role, but synergistic interactions with other molecules cannot be excluded.

## 5. Conclusions

In light of these preliminary findings, the obtained vesicular formulations, particularly those containing glycerol and/or sodium hyaluronate, demonstrated promising dual benefits for treating herpetic sores. On the one hand, they support wound healing and reduce local oxidative damages and inflammation by modulating MMPs and pro-inflammatory cytokines, which are crucial for tissue repair and regeneration. On the other hand, they were able to exert a direct antiviral effect against HSV-1, providing an additional layer of therapeutic efficacy. Overall results highlight the potential of glycerosomes, hyalurosomes, and glycerohyalurosomes as promising delivery systems that enhance the antiviral potential of *L. angustifolia* extract against HSV-1 and may serve as effective alternatives or adjuncts to conventional antiviral therapies. Further studies are needed to evaluate the pharmacological efficacy, skin penetration, and safety of the formulations in more physiologically relevant models, such as ex vivo human skin and in vivo dermal infection models. Moreover, in-depth mechanistic studies, including viral replication kinetics and RT-PCR analysis, will be performed to better elucidate how they interfere with HSV-1 infection pathways.

## Figures and Tables

**Table 1:** Composition of the *Lavandula angustifolia* Miller extract loaded vesicles.

	*L. angustifolia* Extract mg/mL	S75 mg/mL	Glycerol mL	Sodium Hyaluronate Solution 0.2% mL	Sodium Hyaluronate Solution 0.4% mL	Water mL
Liposomes	20	120	-	-	-	1
Glycerosomes	20	120	0.5	-	-	0.5
Hyalurosomes	20	120	-	1	-	
Glycerohyalurosomes	20	120	0.5	-	0.5	

**Table 2:** Mean diameter (MD), polydispersity index (PI) and zeta potential (ZP) of *Lavandula angustifolia* Miller extract loaded vesicles. Each value represents the mean ± standard deviation of at least three replicates and independently repeated in at least three separate batches. The same letter (a, b, and c) indicates values not statistically different from each other (*p* > 0.05) and different from other values (*p* < 0.05).

	MD (nm)	PI	ZP (mV)	EE (%)
Liposomes	74 ^a^ ± 2	0.16	−23 ± 8	100 ^c^ ± 2
Glycerosomes	58 ± 3	0.24	−45 ^b^ ± 4	94 ^c^ ± 6
Hyalurosomes	77 ^a^ ± 3	0.21	−43 ^b^ ± 8	100 ^c^ ± 1
Glycerohyalurosomes	80 ^a^ ± 12	0.27	−42 ^b^ ± 4	54 ± 8

**Table 3:** Antiviral efficacy of *L. angustifolia* extract in dispersion or loaded into formulations against HSV-1 infection in comparison to acyclovir. Data are reported as the half-maximal effective concentration (EC50, µg/mL), and the symbol - indicates data not applicable.

	EC50 (µg/mL)		
	Pre-Infection	During Infection	Post-Infection
Dispersion	-	<0.20	-
Liposomes	-	80.96	-
Glycerosomes	23.82	<0.20	3.53
Hyalurosomes	53.76	1.79	5.76
Glycerohyalurosomes	-	0.59	4.72
Acyclovir	-	9.80	1.91

## Data Availability

Data are contained within the article.
